# Diphtheria and Tetanus Immunity Status among Greek Adults: Results from a Nationwide Seroprevalence Study

**DOI:** 10.3390/vaccines12040378

**Published:** 2024-04-02

**Authors:** Dimitrios Papagiannis, Eleftherios Thireos, Anargiros Mariolis, Antonios Katsioulis, Ioannis Ch. Lampropoulos, Ioanna Tsiaousi, Kostantina Gartzonika, Niki Malliaraki, Foteini Malli, Erasmia C. Rouka, Georgios Marinos, Emmanouil K. Symvoulakis, Georgios Rachiotis, Konstantinos I. Gourgoulianis

**Affiliations:** 1 Public Health & Vaccines Laboratory, Faculty of Nursing, School of Health Sciences, University of Thessaly, 41110 Larissa, Greece; akatsioul@uth.gr (A.K.); errouka@uth.gr (E.C.R.); 2 National Health System of Greece, Primary Health Center of Vari, 16672 Athens, Greece; ethireos@gmail.com; 3 National Health System of Greece, Primary Health Center, 23062 Areopolis, Greece; amariolis@gmail.com; 4 Respiratory Disorders Lab, Faculty of Nursing, University of Thessaly, 41110 Larissa, Greece; i.ch.lampropoulos@gmail.com (I.C.L.); mallifoteini@yahoo.gr (F.M.); 5 Private Primary Health Sector, Queen Sophia Avenue 123, 11521 Athens, Greece; ioannatsiaousi@yahoo.gr; 6 Microbiology Department, Faculty of Medicine, School of Health Sciences, University of Ioannina, 45110 Ioannina, Greece; kgartzon@uoi.gr; 7 Laboratory of Clinical Chemistry-Biochemistry, University Hospital of Heraklion, 71003 Crete, Greece; 8 Department of Hygiene, Epidemiology and Medical Statistics, School of Medicine, National and Kapodistrian University of Athens, 11527 Athens, Greece; gmarino@med.uoa.gr; 9 Department of Social Medicine, Faculty of Medicine, University of Crete, 71003 Heraklion, Greece; esymvoulakis@uoc.gr; 10 Department of Hygiene and Epidemiology, Medical Faculty, School of Health Science, University of Thessaly, 42200 Larissa, Greece; grachiotis@gmail.com; 11 Department of Respiratory Medicine, Faculty of Medicine, School of Health Sciences, University of Thessaly, BIOPOLIS, 41110 Larissa, Greece; kgourg@uth.gr

**Keywords:** tetanus, diphtheria, immunity status, vaccines, booster vaccinations

## Abstract

Diphtheria and tetanus could lead to serious morbidity. We aimed to evaluate immunity levels by measuring specific IgG antibodies for diphtheria and tetanus in serum samples from a nationally expanded sample of the Greek population. A geographically stratified sampling approach based on regional units (NUTS level 2) was applied by considering variables such as age group (30–80+) and sex. In total, 1201 persons (47.7% males and 52.3% females) participated in the survey. Bivariate analysis revealed a negative relationship between diphtheria and tetanus median antibody titers and age. The overall seropositivity rate for diphtheria IgG antibodies (≥0.10 IU/mL) was estimated at 31.5%. Regarding tetanus, the total seropositivity rate was estimated at 59.5% (tetanus IgG antibodies ≥0.10 IU/mL). Logistic regression analysis indicated that age groups <40 years and 40–59 years were independently associated with tetanus seropositivity. Logistic regression also revealed that male sex and being aged 60–69 years were independent risk factors for diphtheria-related seropositivity. Lastly, being resident of some regions was an independent risk factor for both diphtheria- and tetanus-related seropositivity. The present study shows that Greek adults are still not completely immune to diphtheria and tetanus. It is likely possible to achieve optimal immunization coverage by implementing serviceable public health initiatives after comprehending real community needs.

## 1. Introduction

The bacterium Corynebacterium diphtheriae (*C. Diphtheriae*) may cause a severe disease, and this disease is vaccine preventable. Humans are the only reservoir for *C. Diphtheriae* [1]. Diphtheria was a frequent source of sickness and mortality in the days before vaccinations were available. The disease’s epidemiology was altered by the implementation of widespread baby vaccination campaigns, and a notable decline in prevalence was seen globally [2].

According to the European Centre for Disease Prevention and Control (ECDC), 281 cases were reported in Europe in 2022. More specifically, the majority of cases were in Germany (206), though cases also occurred in Belgium (37), Czechia (10), Slovakia (9), the Netherlands (9), Sweden (5), Latvia (3), Norway (1) and Spain (1). Furthermore, two deaths, reported in 2023, occurred in Belgium and Latvia [3]. Greece has been proclaimed diphtheria free since 1994 since the last autochthonous case of the disease was detected in 1982 [4,5]. The latest recorded case of diphtheria occurred in Greece in November 2019 and affected an 8-year-old boy who had pulmonary hypertension and mosaic Down syndrome as underlying disorders [5].

Clostridium tetani (*C. tetani*) toxigenic strains are the source of the acute infectious illness tetanus. The environment is full of *C. tetani* spores, which can enter the body through tissue damage such as puncture wounds or infected skin wounds. The disease can strike at any age, and, even in cases where intensive care is provided, it has a high case fatality rate at any age [6,7]. A large percentage of tetanus occurrences globally are related to birth or delivery. These cases typically occur in low-income nations and are typically connected with unvaccinated women and their newborn children after unsanitary births or abortions, as well as because of inadequate postnatal hygiene practices [8]. For the year 2018, twenty-six member countries of the EU reported ninety-two tetanus cases, of which half were labeled as confirmed, and Italy accounted for nearly half of all registered cases [9].

The incidence of this dangerous disease has significantly decreased as a result of tetanus vaccination. Tetanus-related deaths have decreased by 99% from the time before vaccinations. During the period 2004–2021, the number of tetanus cases reported through the mandatory notification system in Greece was ninety-three. The notification rate during the period 2004–2021 ranged between 0.02/100,000 population and 0.10/100,000 population, while the mean rate over the period 2004–2021 was 0.05 cases per 100,000 population [9]. Elimination of environmental exposure to tetanus is impossible, especially for some people like farm workers. Vaccination against tetanus has been assessed as the most effective intervention. Furthermore, the disease of tetanus is notably resource consuming. The average direct cost of care for tetanus patients is estimated to be substantial in the USA [10]. In addition, residents of low- and middle-income countries are at a higher risk of neonatal tetanus, particularly in locations like rural and urban slums where sanitary home births are widespread and maternal tetanus toxoid immunization and antenatal care service coverage are typically insufficient [11,12]. Tetanus has also a high fatality rate within the adult population. As a systematic review reported, the fatality rate is estimated to be 45.5% in African countries [13].

Many studies have been carried out to estimate the adult population’s vaccination coverage against tetanus and diphtheria in Greece [14,15], but very little is known about the seroprevalence, particularly at a national level [16]. Incidence in less developed countries also decreased after the launch of the World Health Organization (WHO) Expanded Programme on Immunization in 1974. The vaccination coverage of the first three doses for both diseases in Greece approaches 99% in infants [17]. There are differences in the Greek immunization schedule, number of doses given overall and type of vaccine used and included in the program. The Greek national healthcare system covers the cost of the vaccines included in the standard childhood immunization program, as well as booster doses for adults. The Greek immunization schedule is recommended and not mandatory [18].

In addition, information on the regional distribution of diphtheria- and tetanus-related seroprevalence for adults in Greece is not available. Consequently, there is a sparsity of relevant data on the seroprevalence of diphtheria and tetanus among the Greek general population.

In the present study, we aimed to investigate the immune responses to diphtheria and tetanus in Greek adults by using ELISA to detect specific IgG antibodies resistant to diphtheria and tetanus.

## 2. Materials and Methods

### 2.1. Sampling Method

Details on the methodology and the sampling methods have been previously published [19]. In brief, a geographically stratified sampling plan was based on the distribution of the population within the thirteen (13) regions of Greece (Eurostat’s NUTS-2). This sampling plan was applied in order to produce a representative sample, taking into consideration the proportions of specific age groups in the Greek general population (30–39, 40–49, 50–59, 60–69, 70–79 and 80+ years) and sex. The required minimum sample size was determined to be 1100 participants based on the assumption of an expected prevalence of 50% for tetanus and diphtheria antibodies, with a precision of ±3%, a confidence level of 95% and a power of 80% [20]. In the present study, we were able to report data from all 13 regions of Greece, including the South Aegean region. The samples were derived from healthy individuals who visited health facilities for routine screening and reasons unrelated to both diseases. The vaccination status for diphtheria and for tetanus was not taken as a criterion for participants in this study. Exclusion criteria were age under 30 years old and residence and symptomatic status. Each prospective participant was informed about the aims and procedures of the study and could then freely choose whether to participate or not. During September 2021 to March 2023, blood samples were collected from a nationwide network of general practitioners (GPs), including microbiological laboratories of private and public hospitals and primary healthcare facilities. The research protocol complied with ethical standards described in the Declaration of Helsinki [21] and received approval from the ethical committee of the University of Thessaly (protocol number 49/4 June 2021).

### 2.2. Laboratory Examination

The enzyme-linked immunosorbent assay (ELISA) was employed for measuring diphtheria and tetanus antibodies from serum samples [22,23]. We used the SERION ELISA with 99% Sensitivity and Specificity (Institute Virion/Serion GmbH Würzburg, Würzburg, Germany), diphtheria IgG test and tetanus IgG test for detection of human IgG antibodies in serum directed against tetanus and diphtheria toxin. Antibody activity test results were measured in international units (IU)/mL. A level of tetanus antibodies <0.01 IU/mL was defined as offering “no immune protection or seronegative”, an antibody level between 0.01 and 0.1 IU/mL was marked as offering “basic protection or low seropositivity” and a level ≥0.1 IU/mL was defined as offering “full protection” [24]. Diphtheria IgG antibody levels <0.01 IU/mL were considered as offering seronegative susceptibility, levels of 0.01–0.099 IU/mL were considered as providing “basic protection” and levels above 0.1 IU/mL were considered to offer “full protection” against diphtheria [25,26].

### 2.3. Statistical Analysis

Variables were checked for normality of distribution (application of Kolmogorov–Smirnoff test). Medians with interquartile range (1st quartile–3rd quartile) were presented. Absolute and relative frequencies were used. A Chi-squared test or Fisher’s exact test was used for the univariate analysis of prevalence of diphtheria and tetanus seropositivity by sex, age group and region of residence. A Mann–Whitney test or Kruskal–Wallis test was performed to reveal any differences among groups of non-normally distributed quantitative variables. Pairwise comparisons were used for age groups for both endpoints (diphtheria and tetanus seropositivity). Two models of logistic regression analysis were adopted in order to explain risk factors independently associated with the likelihood of seropositivity for both endpoints (diphtheria and tetanus seropositivity).

Logistic regression analysis was performed using the Wald test. Sex, age group and region of residence were included as independent variables (factors) and diphtheria or tetanus antibody levels as dependent variables. The level of statistical significance was set at *p*-value < 0.05. All analyses were performed using IBM SPSS Statistics software version 29.0 (IBM Corp., Armonk, NY, USA).

## 3. Results

**Figure 1:**
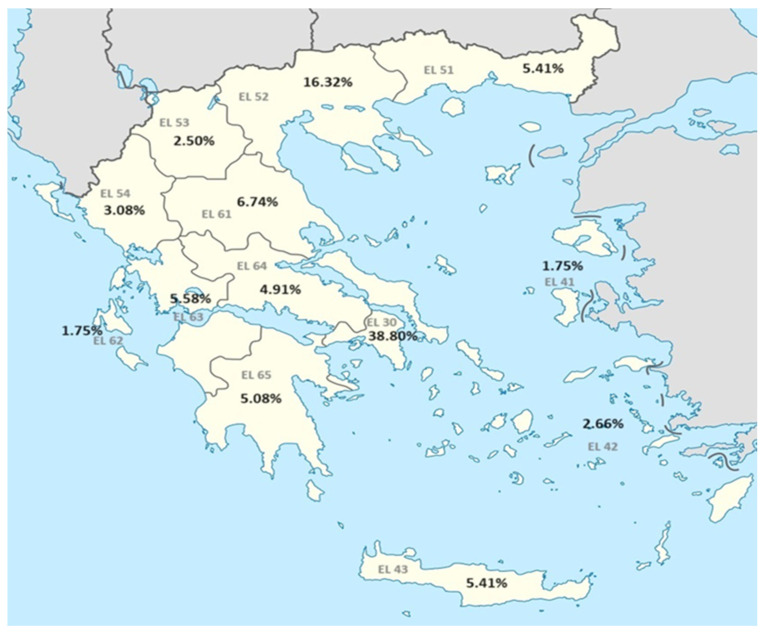
Country map—sample distribution.

Of the 1201 total participants from the 13 regional districts in Greece (NUTS level 2 districts), 573 were male (47.7%) and 628 were female (52.3%) (Table 1). The levels for IgG tetanus antibodies ranged from 0.01 IU/mL to 1.31 IU/mL, with median 0.20 IU/mL and IQR 0.03–0.53 IU/mL, and, for diphtheria, IgG antibodies ranged between 0.00 IU/mL and 0.69 IU/mL with median 0.05 IU/mL and IQR 0.02–0.13 IU/mL. Regarding the sample distribution, in accordance with the census of 2011, most participants originated from the region of Attica (38.80%), while participants from North Aegean and Ionian Islands accounted for 1.75% (Table 1, Figure 1).

We recorded that the estimated overall seroprotective rate for diphtheria IgG antibodies ≥0.10 IU/mL was 31.5%. We report differences among age groups, with the age groups <40 having the highest prevalence of protective antibodies for diphtheria in comparison to older groups (*p*-value = 0.023) in both sexes. Regarding tetanus, we recorded the total seroprotective rate for 715 participants (59.5%) with tetanus IgG antibodies ≥0.10 IU/m. As previously reported for diphtheria, the age groups <40 exhibited significant differences in comparison to the rest of the age groups in terms of having seroprotective antibodies against tetanus (*p*-value < 0.001) (Table 2). 

Of the total 573 male participants, 201 (35.1%) had seroprotective titers against diphtheria in comparison to 177 females (28.2%) (*p*-value = 0.010). Slight differences for protective antibodies were recorded for tetanus among male and female subjects. Specifically, 357 men (62.3%) had protective antibodies ≥0.10 IU/m in comparison to 358 females (57%) (*p*-value = 0.062) (Table 2).

**Figure 2:**
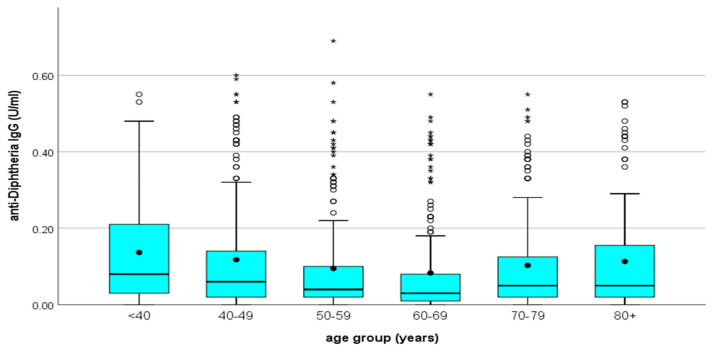
Distribution of IgG diphtheria antibody titers by age group. **^O^** outliers. * extreme outliers. • mean.

**Figure 3:**
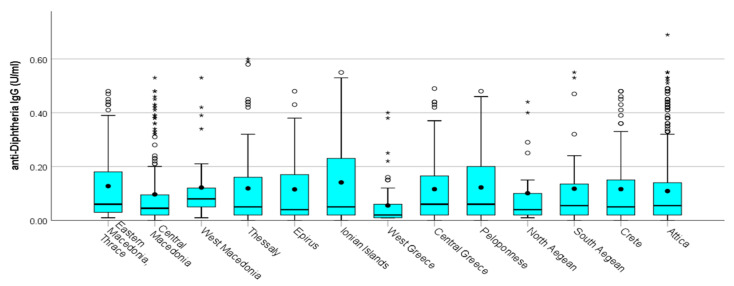
Distribution of IgG diphtheria antibody titers by region. **^O^** outliers. * extreme outliers. • mean.

The majority of diphtheria IgG antibodies varied between 0.001 and 0.20 IU/mL. IgG antibodies protective against diphtheria were highest in the age groups <40, and the lowest proportion was recorded in the age group >80 (Table 2) (Figure 2). IgG antibodies decreased by age group. Significant differences in antibody levels were found between the younger (30–49 years) and the oldest (60–80 years) participants (Figure 3). This observation was more pronounced in regions with commonly low antibody levels, such as West Greece, which had a mean concentration of 0.06 IU/mL, in comparison to, for example, Eastern Macedonia and Thrace with 0.13 IU/mL and Ionian Islands with 0.14 IU/mL. We recorded the highest proportion of antibodies <0.01 IU/mL in the age group of 60–69, with a total proportion of 24.90%, followed by the age group of 70–79 with 19.80%. 

**Figure 4:**
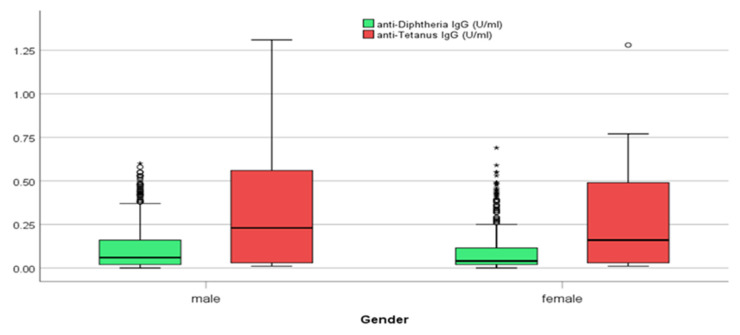
Frequency of IgG antibodies for diphtheria and tetanus by gender. **^O^** outliers. * extreme outliers.

Diphtheria IgG antibodies were significantly higher (*p*-values = 0.002) in males (median = 0.06 IU/mL with IQR: 0.02–0.16 IU/mL) in comparison with females (median = 0.04 IU/mL with IQR: 0.02–0.12 IU/mL) (Mann–Whitney test) (Figure 4). The median concentration values for IgG diphtheria declined as the age increased for all regions and both sexes (Figure 2 and Figure 4).

### 3.1. Univariate Analysis

Univariate analysis found that diphtheria and tetanus seropositivity was significantly associated with male gender. This also was the case for age. Furthermore, bivariate analysis detected a negative relationship between diphtheria and tetanus median antibody titers (Spearman’s correlation coefficient for diphtheria mean antibody titers and age = −0.102, *p* < 0.001; Spearman’s correlation coefficient for tetanus-related median titers and age = −0.210, *p* < 0.001). With respect to the distribution of diphtheria and tetanus median antibody titers by region, we report a considerable variation for both outcomes (*p* < 0.001 for tetanus-related antibodies, and *p* = 0.0062 for diphtheria; Kruskal–Wallis test).

**Figure 5:**
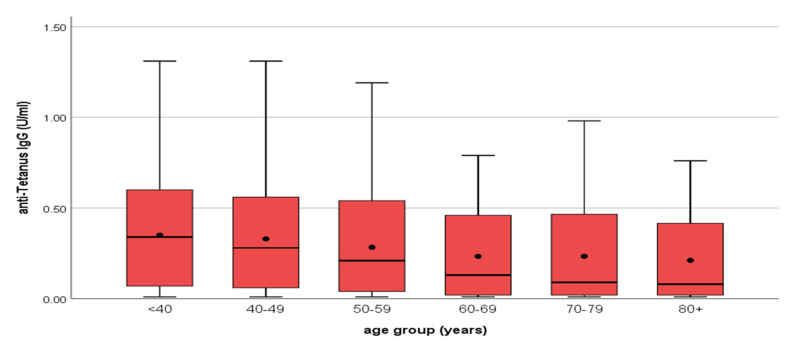
Distribution of IgG tetanus antibody titers by age group. • mean.

**Figure 6:**
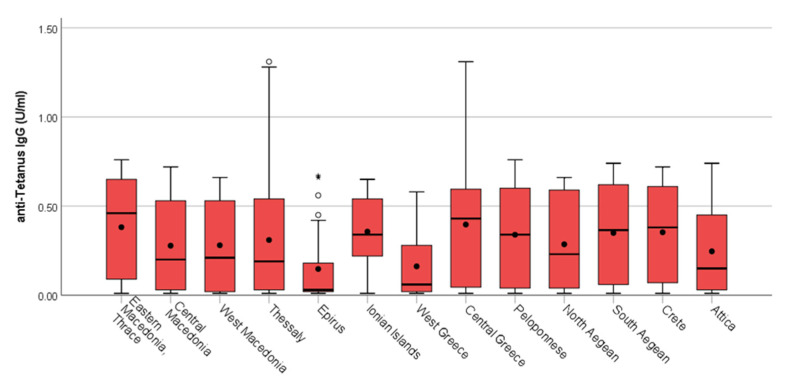
Distribution of IgG tetanus antibody titers by region. **^O^** outliers. * extreme outliers. • mean.

Tetanus IgG antibody titers <0.01 IU/mL were considered seronegative. When considering the antibodies for tetanus, we recorded that 92 out of the 1201 samples were under the cut off of 0.01 IU/mL, and the total proportion of sera with levels below 0.01 IU/mL was 7.6%. The vast majority of tetanus IgG antibodies varied between 0.001 and 0.20 IU/mL. Tetanus mean IgG antibody titer values were usually increased in men versus women. Tetanus IgG antibodies were significantly higher (*p*-values = 0.009) in males (median = 0.23 IU/mL with IQR: 0.03–0.56 IU/mL) in comparison with females (median = 0.16 IU/mL with IQR: 0.03–0.49 IU/mL) (Mann–Whitney test) (Figure 4). As reported previously for the diphtheria antibodies, significant differences in mean tetanus antibody titer levels were found between the younger (30–59 years) and older (60–80 years) participants for tetanus too (Figure 5). The regions with the lowest mean titer of IgG antibodies were the regions of Epirus and West Greece, and the regions with the highest were the regions of Eastern Macedonia and Thrace followed by the region of Central Greece (Figure 6).

**Figure 7:**
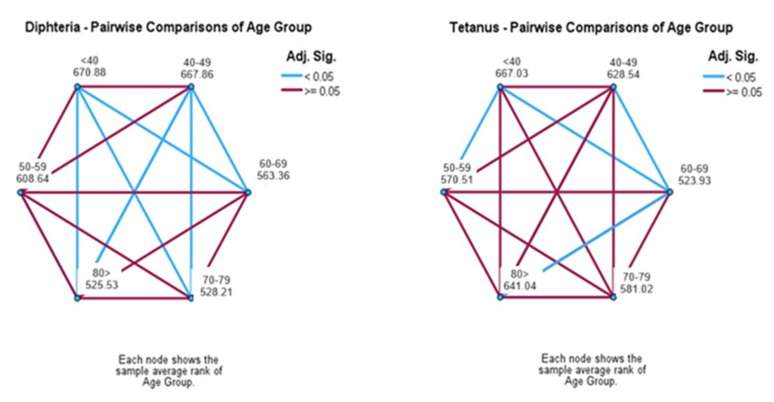
Pairwise age group comparisons and IgG antibodies for tetanus and diphtheria.

In pairwise comparisons between age groups, the younger ages had the highest titers of IgG antibodies against both diseases. Especially for diphtheria, age groups <40 and 40–49 exhibited significant differences in comparison to the 60–69, 70–79 and 80+ age groups (Figure 7). Similar results were recorded for the age groups <40 for tetanus antibodies in comparison to the 50–59 and 60–69 age groups. Significant differences were recorded for the age group of 60–69 compared with participants with age >80 (Figure 7).

### 3.2. Logistic Regression Analysis of Diphtheria- and Tetanus-Related Seropositivity

Logistic regression analysis has shown that men were more likely to be positive for diphtheria antibodies (OR = 1.39; 95% CI: 1.09–1.79; *p* = 0.009) than women. The age group of 60–69 years was significantly less likely to be positive for diphtheria antibodies compared to the age group of 80+ years (OR = 0.49; 95% CI: 0.30–0.80; *p* = 0.005). The Central Macedonia region was significantly less likely than the Attica region to be associated with positivity for diphtheria antibodies (OR = 0.61; 95% CI: 0.42–0.91; *p* = 0.014). In addition, the West Greece region was significantly less likely than the Attica region to show a considerable prevalence of diphtheria antibodies (OR = 0.35; 95% CI:0.18–0.70; *p* = 0.003) (Table 3).

### 3.3. Seropositivity of Antibodies Specific to Tetanus Toxoid

According to the logistic regression results, gender was not an independent predictor of antibody seropositivity related to the specific tetanus toxoid (OR = 1.26; 95% CI: 0.99–1.60; *p* = 0.058). The age groups >40 years were significantly more likely than the age group 80+ years to be positive for antibodies specific to the tetanus toxoid (OR = 2.73; 95% CI: 1.72–4.33; *p* < 0.001). The age group of 40–49 years was more likely than the age group of 80+ years to be positive for specific tetanus toxoid antibodies (OR = 2.90; 95% CI: 1.86–4.54; *p* < 0.001). Additionally, the age group of 50–59 years was significantly more likely than the age group of 80+ years to be positive for tetanus antibodies (OR = 1.56; 95% CI: 1.01–2.43; *p* = 0.045). Those from the Epirus region were significantly less likely than those from the Attica region to be positive for tetanus antibodies (OR = 0.43; 95% CI: 0.21–0.87; *p* = 0.019). Additionally, those from West Greece were significantly less likely than those from Attica to be positive for tetanus antibodies (OR = 0.38; 95% CI: 0.22–0.66; *p* = 0.001), as shown in Table 4.

## 4. Discussion

Despite the high vaccination coverage in infants and young children in Greece, the present study demonstrates that a substantial portion of participants remain susceptible to vaccine-preventable diseases such as diphtheria and tetanus. In the present nationwide seroprevalence study, we present results with an overall positivity rate for IgG antibodies against tetanus in an adult population group in Greece different from that in a previous study conducted across European countries including Greece [16]. These differences may be related to differences in the methodological approaches between the two studies in terms of the geographical aspects of sample selection and different demographic background (e.g., age) of the participants. Our results differ from those of a study in Italy, which reported that a significant minority (22%) of Italian construction workers were inadequately protected against tetanus [27].

Determining how long vaccine-induced immunity lasts is essential for making well-informed decisions on the optimal interval between booster shots. Tetanus IgG antibodies decreased with an eleven-year half-life in longitudinal trials with a small number of participants [28]. We present results that indicated a reduction in the mean titer of antibodies by age group. The results for the immunity status of the adults of the present study reflectively support data published by the National Public Health Organization (EODY) about the age and gender distribution of tetanus incidence. Especially for the period 2004–2021, the disease presented the highest frequency of occurrence in the age group >65 years old, with a mean annual notification rate of 0.16 cases per 100,000 population. This notification rate is higher than that of the other age groups (5–14, 25–44 and 45–64), in which it did not exceed 0.04 cases per 100,000 population. No cases have been reported in children below the age of 4 years old, and these data are supported by the high vaccination rates for this age. The mean annual notification rate for men was equal to that for women (0.05/100,000 population) [9]. Of the 93 cases that were documented between 2004 and 2021, 58 patients (62.4%) had not ever had a vaccination. Only ten cases—four with one dose of the vaccine, two with two doses and one with three doses—were reported to have had tetanus vaccinations according to the EODY’s analysis of the data. In four cases, there was no information available regarding the number of doses administered. There was no information provided regarding the vaccination status in 23 instances. Most of the cases, particularly in the over-65 age group, were unvaccinated [9].

With the passing of years, the immunological response to the tetanus vaccination seems to diminish. Comparative research has shown that adolescents often produce higher antibody levels than adults. Most adult immunizations contribute and sustain protective levels of antibodies for many years despite the decline in immunogenicity. The importance of booster doses has been supported by evidence from many studies in the past. American soldiers received booster shots after wounds in addition to two or three rounds of the primary vaccine series [10]. Out of twelve million injured US army participants, only twelve cases of tetanus were reported [10]. Soldiers across the board (0.44 per 100,000) were compared to 70 of the 520,000 injured in the Second World War (13.4 per 100,000), and only four of the twelve had received all of their main vaccinations [11]. The data of the present study support the necessity for booster vaccinations against tetanus in Greek adults and indicate that an important proportion of the Greek adult population is susceptible. Many studies have repeatedly reported insufficient antibody concentrations against both tetanus and diphtheria (<0.1 IU/mL) in adults, particularly in the elderly [19,20,21,22,23,24,25,26,27,28,29,30,31]. Our results agree with previous studies that the concentration of protective antibodies decreases with increasing age.

Regarding immunity against diphtheria, we recorded a large proportion of participants with limited protection against the disease (67.3%), while 31.50% had antibodies within the protective level. Similar results for diphtheria, showing a low level of protection, were reported for Greece by a European study conducted six years before the present study [32]. Results from a study among immunized healthy Slovak adults assessed the long-term pattern of humoral immunity in the case of diphtheria, with only 21% of individuals found to have seroprotective antibodies ≥0.1 IU/mL before the booster immunization [33].

Completely vaccinated individuals seldom catch the disease, and even when they do, outbreaks in communities highly exposed to the toxin-producing C. diphtheriae are uncommon [34]. However, illness in those who have received vaccinations is typically moderate, with fewer sequelae and no fatalities. We present data showing that, in most regions, diphtheria-specific IgG antibodies decreased by age group. People between the ages of 40 and 59 had inadequate levels of seroprotection against diphtheria according to recent seroprevalence research carried out in 16 European nations [16]. This emphasizes how crucial it is to receive booster shots containing the diphtheria toxoid after the immunization campaign. Adults should receive booster doses at different intervals, and it is critical to ensure that everyone is vaccinated against diphtheria. Results for these special age groups reported in a previous study in Greece showed very low vaccination coverage for tetanus and diphtheria [14].

Another finding of this cross-sectional seroprevalence study (secondary analysis data) was the significant differences in antibody levels between the age cohorts when comparing the younger (30–49 years) to the older (60–80 years) participants. A study in Vietnam found significant variation between the age groups of 21–30 and 31–40 compared to the age group of 60+ years (*p* < 0.05) [35]. In countries where routine vaccination rates are low, diphtheria is nevertheless endemic. The regular repetition of booster immunizations can help to sustain full protection of the population and enhance the herd immunity. The outbreak in the former Soviet Union in the recent past was caused by a number of circumstances, including a lack of population immunity, a weak socioeconomic infrastructure and a delayed public health response [36,37]. Greece has a low overall incidence and a high childhood immunization rate against tetanus and diphtheria toxoids [4,5]. Compared to booster vaccinations for the adult population [14], Greece has a higher vaccination rate for newborns [3], including both the first and third doses of the diphtheria, tetanus toxoid and pertussis vaccines.

The WHO reports that preschool-age and school-age children are most frequently vulnerable to diphtheria when they are either unvaccinated or have not received enough vaccinations. The amount of diphtheria antitoxin obtained and the length of protection are influenced by the formulation of the diphtheria toxoid and the timing of vaccination delivery [38]. Regarding the tetanus toxoid, the ability to evoke booster responses even after intervals of 20 years or more shows that immunological memory is persistent. Data from serological surveys indicate that adults and teenagers who want to maintain high antibody levels that can last for decades must take booster doses [39]. The American guideline panel on vaccination practices, the Advisory Committee on Immunization Practices, advises the Centers for Disease Control and Prevention to prescribe booster doses for adults every ten years [40].

It is believed that a significant factor contributing to the increased incidence and prevalence of chronic illnesses such as metabolic, neurological and cardiovascular disorders is the aging immune system, or immunosenescence. These illnesses frequently manifest clinically in the elderly population as multimorbidity, which raises the risk of organ failure and mortality. As immunosenescence progresses, older persons are also more vulnerable to infectious infections. In practical terms, this means that vaccination schedules should be modified to account for the immunological responses that are observed in clinical studies with elderly participants.

The published research demonstrates that the two sexes respond differently to immunological stimuli in terms of both innate and adaptive immunity, yet this is not taken into account when developing recommended vaccination schedules for any age. The persistence of gender differences in the innate immune system in older adults has not received enough attention, yet some studies indicate that females continue to produce more inflammatory proteins than males do [41]. In the present study, we recorded gender differences in the level of immunity for both diphtheria and tetanus. Univariate analysis found that diphtheria and tetanus seropositivity was significantly associated with male gender. One explanation is that a booster dose was given to the men on entrance to the military corps. According to the Greek army, vaccinations against tetanus diphtheria and pertussis at the introduction to the army are mandatory, independent of the immunological status of the subjects. Similar results regarding gender and tetanus antibodies are presented by Weinberger et al. This is probably due to vaccination during military service or more frequent booster vaccinations after injuries [32].

The present study has several limitations. Taking into consideration that our sample was based on a non-probability sampling method, we may underline that the seroprevalence figures from the present sample of participants might not represent the prevalence of tetanus and diphtheria for the whole country. It is difficult to discern between the particular humoral immune responses brought on by vaccinations and those brought on by infection. Furthermore, it was unknown whether the subjects had received any vaccinations. Another limitation of the study is that we did not include immigrants in the population study. The migrants leaving countries with poor immunization programs or where vaccinations have been interrupted may represent a new risk group in host countries. A study conducted in six European countries depicted that 22.3% of the participants were without protective antibodies for tetanus [42].

Despite these drawbacks, our study has the advantage of being the first to offer some estimates of the nationwide seroprevalence of tetanus and diphtheria IgG antibodies among adults in Greece using stratified sampling, and the findings may be useful as recently reported information for policymakers and health professionals in planning future campaigns to prevent these diseases.

## 5. Conclusions

The present study shows that Greek adults are still not longitudinally immune to diphtheria and tetanus. It is likely that optimal immunization coverage could be achieved by implementing sensible public health initiatives. In order to maintain protection into old age, these measures would need to incorporate a lifelong perception through public health and primary-care-driven education on vaccinations as repeated booster shots throughout maturity are required. Our study findings about regional differences serve as a call for uniform vaccination incentives throughout Greece. Apart from personalized motivational support, enhancing public awareness of the advantages and disadvantages of vaccinations could potentially boost adherence to immunization protocols. Generally, antibodies were higher in some regions than others, but, interestingly, variations between regions were not proportionally similar. The local vaccination policies followed by the physicians of primary healthcare or occupational health settings could be an explanation for this variation. This is surprising because combo vaccinations are advised to prevent both tetanus and diphtheria. Despite the prescription for combined immunizations, it appears that this suggestion is not always followed, and it is possible that solo tetanus vaccinations are occasionally used.

## Figures and Tables

**Table 1:** Demographic background of the participants.

Variable	N	%
Sex		
Male	573	47.7%
Female	628	52.3%
Total	1201	100%
Age Group		
<40	222	18.50%
40–49	226	18.80%
50–59	214	17.80%
60–69	209	17.40%
70–79	191	15.90%
80+	139	11.60%

**Table 2:** Univariate analysis of IgG seroprotective diphtheria and tetanus antibodies.

	Diphtheria IgG (U/mL) (Two Groups)							Tetanus IgG (U/mL) (Two Groups)					
		Count 0.10+	%	Count <0.10	%	Total	*p*-Value *	Count 0.10+	%	Count 0.01–0.09	%	Total	*p*-Value *
Sex	Male	201	35.1%	372	64.9%	573	0.010	357	62.3%	216	37.7%	573	0.062
	Female	177	28.2%	451	71.8%	628		358	57.0%	270	43.0%	628	
Age	<40	87	41.6%	122	58.4%	209		149	71.3%	60	28.7%	209	<0.001 **
	40–49	84	35.3%	154	64.7%	238	0.023 **	172	72.3%	66	27.7%	238	
	50–59	54	25.2%	160	74.8%	214		127	59.3%	87	40.7%	214	
	60–69	44	21.1%	165	78.9%	209		108	51.7%	101	48.3%	209	
	70–79	59	30.9%	132	69.1%	191		92	48.2%	99	51.8%	191	
	80+	49	35.3%	90	64.7%	139		66	47.5%	73	52.5%	139	
Region	Eastern Macedonia, Thrace	24	36.9%	41	63.1%	65	0.180	47	72.3%	18	27.7%	65	0.001
	Central Macedonia	49	25.0%	147	75.0%	196		122	62.2%	74	37.8%	196	
	West Macedonia	11	36.7%	19	63.3%	30		20	66.7%	10	33.3%	30	
	Thessaly	30	37.0%	51	63.0%	81		46	56.8%	35	43.2%	81	
	Epirus	12	32.4%	25	67.6%	37		15	40.5%	22	59.5%	37	
	Ionian Islands	8	38.1%	13	61.9%	21		16	76.2%	5	23.8%	21	
	West Greece	11	16.4%	56	83.6%	67		26	38.8%	41	61.2%	67	
	Central Greece	23	39.0%	36	61.0%	59		38	64.4%	21	35.6%	59	
	Peloponnese	22	36.1%	39	63.9%	61		41	67.2%	20	32.8%	61	
	Attica	152	32.6%	314	67.4%	466		267	57.3%	199	42.7%	466	
	North Aegean	6	28.6%	15	71.4%	21		11	52.4%	10	47.6%	21	
	South Aegean	10	31.3%	22	68.8%	32		21	65.6%	11	34.4%	32	
	Crete	20	30.8%	45	69.2%	65		45	69.2%	20	30.8%	65	

* Chi-squared test. ** Chi-squared test for trend. Pearson Chi-squared tests.

**Table 3:** Multivariate analysis of diphtheria IgG (≥0.10 U/mL).

Factors	Odds Ratio	95% CI	*p*-Value
Sex Male	1.39	1.09–1.79	0.009
Female	ref.		
Age group <40	1.42	0.90–2.24	0.135
40–49	1.06	0.68–1.65	0.802
50–59	0.63	0.40–1.02	0.059
60–69	0.49	0.30–0.80	0.005
70–79	0.83	0.52–1.33	0.447
80+	ref.		
Region Eastern Macedonia, Thrace	1.12	0.64–1.94	0.695
Central Macedonia	0.61	0.42–0.91	0.014
West Macedonia	1.10	0.50–2.40	0.815
Thessaly	1.15	0.70–1.89	0.585
Epirus	0.91	0.44–1.88	0.791
Ionian Islands	1.17	0.47–2.92	0.744
West Greece	0.35	0.18–0.70	0.003
Central Greece	1.22	0.69–2.16	0.490
Peloponnese	1.04	0.59–1.84	0.890
North Aegean	0.78	0.29–2.09	0.625
South Aegean	0.84	0.38–1.85	0.664
Crete	0.82	0.46–1.46	0.506
Attica	ref.		

The reference category is <0.10 IU/mL.

**Table 4:** Multivariate analysis of tetanus IgG antibodies (≥0.10 IU/mL).

Factors	Odds Ratio	95% CI	*p*-Value
Sex	1.26	0.99–1.60	0.058
Male			
Female	ref.		
Age group	2.73	1.72–4.33	<0.001
<40			
40–49	2.90	1.86–4.54	<0.001
50–59	1.56	1.01–2.43	0.045
60–69	1.16	0.75–1.79	0.509
70–79	0.99	0.63–1.54	0.963
80+	ref.		
Region	1.72	0.95–3.09	0.071
Eastern Macedonia, Thrace			
Central Macedonia	1.04	0.73–1.49	0.811
West Macedonia	1.29	0.58–2.87	0.535
Thessaly	0.89	0.55–1.46	0.654
Epirus	0.43	0.21–0.87	0.019
Ionian Islands	2.13	0.75–6.03	0.154
West Greece	0.38	0.22–0.66	0.001
Central Greece	1.19	0.66–2.12	0.563
Peloponnese	1.35	0.76–2.42	0.307
North Aegean	0.73	0.30–1.79	0.488
South Aegean	1.19	0.55–2.57	0.666
Crete	1.42	0.80–2.52	0.227
Attica	ref.		

The reference category is 0.01–0.09 IU/mL.

## Data Availability

The data that support the findings of this study are available on request from the corresponding author.
